# Family Structure and Cognitive Effort: Examining the Influence of Living with a Single Parent on Children’s Task Engagement

**DOI:** 10.1007/s11577-026-01082-9

**Published:** 2026-06-02

**Authors:** Jonas Radl, Miguel Requena, Madeline Swarr

**Affiliations:** 1 Departamento de Ciencias Sociales, Instituto Juan Linz, Universidad Carlos III Madrid, Getafe, Community of Madrid Spain; 2 Wissenschaftszentrum Berlin für Sozialforschung gGmbH, Berlin, Germany; 3 Departamento de Sociología II, Universidad Nacional de Educación a Distancia, Madrid, Community of Madrid Spain

**Keywords:** Executive function, Effortful behavior, Primary education, Family structure, Early adolescence, Cross-national study, Exekutive Funktionen, Anstrengungsbereitschaft, Grundschule, Familienstruktur, Frühe Adoleszenz, Internationaler Vergleich

## Abstract

This brief report investigates the association between family structure and the cognitive effort of fifth-grade students in Madrid and Berlin. Although existing studies often link single parenthood to poorer academic outcomes, scant research has investigated its impact on children’s effort. This study utilizes a novel behavioral measure of cognitive effort—rather than survey-based proxies—to fill this knowledge gap.

We collected data from 1359 students across 62 classes. Effort was measured via performance on three incentivized real-effort tasks (AX continuous performance, Simon, and slider tasks). Using two-level random-intercept linear regression models and multiple imputation, we analyzed children’s effortful behavior while adjusting for cognitive ability.

Our findings consistently show that children from single-parent households exhibit lower cognitive effort. This negative association remained significant across all incentive conditions and even after controlling for socioeconomic factors and environmental variables. Moreover, the effort gap between single and dual-parent households was qualitatively similar in both cities. However, the estimated magnitude of this effect is rather small.

In conclusion, children growing up with a single parent exhibit lower cognitive effort than children in dual-parent families. This finding may provide a pathway to better understanding of the educational disadvantage typically observed among single-parent children.

## Abstract (de)

Dieser Kurzbericht untersucht den Zusammenhang zwischen der Familienstruktur und der kognitiven Anstrengung von Fünftklässlern in Madrid und Berlin. Während bestehende Studien die Abwesenheit eines Elternteils häufig mit geringerem Schulerfolg verknüpfen, wurde der Einfluss auf die kindliche Anstrengungsbereitschaft bislang kaum erforscht. Um diese Forschungslücke zu schließen, nutzt die vorliegende Studie ein neuartiges verhaltensbasiertes Maß für kognitive Anstrengung – anstelle von umfragebasierten Proxy-Variablen.

Die Datengrundlage umfasst 1359 Schülerinnen und Schüler aus 62 Klassen. Die Anstrengung wurde über die Leistung in drei inzentivierten „Real-Effort“-Aufgaben (AX-, Simon- und Slider-Task) gemessen. Mittels linearer Zwei-Ebenen-Regressionsmodelle und multipler Imputation wurde das Anstrengungsverhalten der Kinder unter Kontrolle ihrer kognitiven Fähigkeiten analysiert.

Unsere Ergebnisse zeigen konsistent, dass Kinder aus Haushalten von Alleinerziehenden eine geringere kognitive Anstrengung aufweisen. Dieser negative Zusammenhang blieb über alle Anreizbedingungen hinweg sowie nach Kontrolle sozioökonomischer Faktoren und Kontextvariablen signifikant. Zudem erwies sich der Unterschied in der Anstrengung zwischen Kindern von Alleinerziehenden und Paarfamilien in beiden Städten als qualitativ ähnlich. Die geschätzte Effektstärke ist jedoch als eher gering einzustufen.

Zusammenfassend lässt sich festhalten, dass Kinder, die bei einem alleinerziehenden Elternteil aufwachsen, eine geringere kognitive Anstrengung zeigen als Kinder aus Paarfamilien. Dieser Befund bietet einen neuen Ansatzpunkt, um die Bildungsbenachteiligung von Kindern Alleinerziehender besser zu verstehen.

## Introduction

Studies abound showing that children who do not live with their two biological parents fare worse on average than those who do live with them (Amato 2005; Härkönen et al. 2017; McLanahan and Sandefur 1994). Parental separation, parental loss, or solo reproduction generate, if there is no new partnering, single-parent families whose children exhibit worse cognitive, behavioral, emotional, and socioeconomic outcomes than children of married couples (Cavanagh and Fomby 2019). An inventory of the so-called absent-parent effect includes negative outcomes in children’s psychological well-being and mental health, family relationships, and employment and economic achievement (Raley and Sweeney 2020). Given the long-term adverse effects of poor educational results, a particular research focus has been the lackluster academic performance of single-parent students (e.g., De Lange, Dronkers and Wolbers 2013; Anthony et al. 2014).

However, there is scant research on the association between single parenthood and children’s cognitive effort, i.e., the mobilization of mental resources for task performance. If single-parent children tend to provide less effort, this would explain frequent findings of their educational underachievement. Although some existing studies report negative correlations with effort-adjacent survey-based personality scales, we lack evidence based on behavioral measures of cognitive effort that eschew the measurement issues characteristic of self-, parent-, or teacher-reported personality (Neuenschwander et al. 2013; Dang et al. 2020). This study aims to contribute empirical evidence on the implications of single parenthood for children’s cognitive effort, key to a better understanding of the diminished school performance of single-parent students.

The absence of one of the parents, usually the father, triggers a chain of interlocking adverse effects that often undermine children’s well-being (Ribar 2015). The first link in the chain is often the stress associated with parental conflicts preceding separation. Some studies relate family crises to changes in children’s basal cortisol levels that point to increased stress (Suor et al. 2015). Others indicate that exposure to family instability hinders children’s responses to emotional conflict and makes them more susceptible to subsequent behavioral problems (Coe et al. 2017). The stress of parental separation may cause more reactive and less reflective behavior, reducing the likelihood of engaging the executive functions (Arnsten 2009; Finegood and Blair 2017). Moreover, living arrangements resulting from couple breakdown are associated with downward income mobility (Bloome 2017) and less disposable household income (Kalmijn et al. 2007; McBride Murry and Lippold 2018). Living with single parents also tends to produce a reduction in social capital following the loss of contact with relatives and friends of the absent parent (Ahrons 2007). Increased family conflict, often linked to recent parental separation, may accompany single parenthood and harm children’s well-being (Demo and Acock 1996).

The depletion of resources in nonintact families frequently causes the quality of parenting to decline, as happens, for example, when the stresses of loss or separation generate emotional instability in the parent caregiver. Parent–child estrangement and diminished paternal supervision and engagement, resulting in adversities in children’s socioemotional development and loss of child well-being, are relatively common experiences among children living with only one parent (Hadfield et al. 2018). The literature on the emotional difficulties of these children is extensive: Poorer socioemotional development (Bzostek and Berger 2017), behavioral problems (Carlson and Corcoran 2001)—in particular, externalizing and internalizing problems (Kim 2011)—and problematic adjustments (Ganong et al. 2015) are more likely in single-parent families than in dual-parent families.

Studies that scrutinize the implications of parental separation for children’s cognitive skills or standardized achievement tests typically find negative effects (Cherlin et al. 1991; Tartari 2015). Similarly, studies examining personality traits, well-being, or sociobehavioral problems tend to find that children living in single-parent families show more adverse characteristics than children in two-parent families (Amato 2005; Sun and Li 2002). However, studies adopting research designs accounting for unobserved confounding sometimes find null effects for certain outcomes (Kim 2011; Garriga and Pennoni 2022).

Of particular interest to our study is previous research on the associations between parental separation and those facets of children’s personality that are conceptually closely related to cognitive effort, such as conscientiousness and locus of control. Smith (1990) reported negative effects of parental separation on academic self-concept, arguably driven by psychological problems arising from trauma. Radl et al. (2017) similarly found a negative association between father absence and math-related locus of control. Further, Anthony et al. (2014) reported that diminished approaches to learning mediate the relationship between parental divorce and academic achievement.

Andrews et al. (2021) showed that household chaos had both a direct effect on child executive functions and an indirect effect via parental responsiveness. However, there are indications that mothers’ involvement is more consequential than fathers’ (Roskam et al. 2014). Moreover, while parental support and discipline were shown to be positively associated with a child’s “effort direction,” i.e., the decision to voluntarily do a real-effort task, inconsistent effects were found for “effort intensity,” i.e., the sustained exertion of cognitive resources (Foley and Radl 2024).

In summary, there is broad consensus in the scientific literature (Amato 2010; Kim 2011; Sun and Li 2002) that the negative implications for children in single-parent families arise from the multiple stressors that tend to accompany—or precede—family disruptions: economic deprivation, family anxieties and their impact on the quality of parenting and socialization, and the uprooting of the child’s life that often comes with the transition to new family configurations. However, we do not know precisely through which mechanisms the stressors triggered by family disruptions translate into poor educational outcomes. As has been pointed out (McLanahan et al. 2013), one of the big pending challenges of this line of research is not so much to estimate the causal effect of parental absence on educational outcomes as to understand how these effects come about. Hence, it is pertinent to examine the cognitive effort provided by children of single parents, a plausible precursor to their poor educational outcomes that has remained underexplored so far.

### The Current Study

In this brief report we document the relationship between living with a single parent and cognitive effort based on an experimental design producing behavioral data among a large and balanced laboratory (in-the-field) sample of fifth-grade students (aged around 11 years) in the urban areas of Madrid and Berlin. Given the large body of research demonstrating that children raised in single-parent families tend to have comparatively poorer educational outcomes, we hypothesized that residing in a single-parent household would be linked to lower levels of cognitive effort, even when other socioeconomic factors are taken into account. Even without knowing what specific processes have led to single parenthood and how long this situation has lasted, it can be assumed that the absence of one parent implies a reduction in emotional, relational, social, and/or economic resources of the family. These shortcomings can trigger family stress (Suor et al. 2015; Coe et al. 2017), impede executive function (Arnsten 2009; Finegood and Blair 2017), and undermine children’s locus of control (Radl et al. 2017). We further explored whether this negative association with cognitive effort might be explained by mechanisms such as reduced parental engagement or increased parental stress or by variations in parenting styles, such as differences in support or discipline.

## Materials and Method

### Participants and Procedure

Both behavioral and survey data were collected from 1359 students representing 62 fifth-grade classes from 35 schools in Madrid and Berlin from October 2019 to February 2022. About 25% of invited schools took part, selected randomly based on neighborhood income and school type (public, private, mixed). Distributions by sampling cluster are shown in Table 2 in the appendix, showing reasonable balance and representativeness of the school systems in each site.^1^ Both children and parents filled out surveys on sociodemographic and personality information. Each child participated only if they had their parent’s or guardian’s written informed consent and signed data protection agreement, in accordance with stipulations of the ethics board and data protection officer at the university. The experiments were implemented using Open Sesame (v.3.2.8; Mathôt et al. 2012).

Behavioral data were collected from three tasks that were adapted from cognitive science tests and behavioral economics “real-effort” experiments and were designed to measure different dimensions of *executive function *(EF), that is, attention-related brain functions that are required for directed cognitive activity. (1) The AX continuous performance task (AX task) measures cognitive flexibility and switching (Hefer and Dreisbach 2016). Participants observed two-letter sequences and pressed a button when any letter other than “A” appeared first or when “A” was followed by any letter other than “X.” They pressed a different button when “A” was followed by “X.” (2) The Simon task tests regulation, inhibition, and control (Cespón et al. 2016). Participants pressed one button for a left-pointing arrow and another for a right-pointing arrow, regardless of arrow position. The task requires inhibiting the reflex to press the left button when, for example, a right-pointing arrow appears on the left side. (3) The slider task assesses working memory, information processing, and updating (Gill and Prowse 2019). Participants adjusted a slider on 48 horizontal lines to the midpoint, corresponding to 50 on a 0–100 scale. In Madrid, tasks were conducted in a university laboratory, while in Berlin, mobile laboratories were set up in schools due to the COVID-19 pandemic.

Participants completed the real-effort tasks under three conditions: first in an unincentivized condition with no rewards for task performance, next under a piece-rate condition with material rewards in which they received points convertible to toys for task performance, and finally in a tournament condition with added status incentives in which the top three performers received a diploma and applause at the final award ceremony (but everyone still earned reward points for performance). The sequence in which tasks were completed varied across experimental sessions to avoid order effects. Before each round, the student could choose to do the task or play the leisure game, but rates of gaming were very low in the incentivized conditions and did not differ between students of single- versus dual-parent households.

### Measures

#### Single-Parent Household

Whether the child lived in a single-parent household was determined by whether the parent or guardian who completed the survey indicated that the child lived with him or her and not with his or her partner or spouse, and vice versa.

#### Cognitive Effort

Cognitive effort was measured by performance scores (total correct answers given per 2‑min round for each of the three tasks described above), standardized across the task-specific distributions of all round scores and adjusted for cognitive ability. This approach follows the emerging consensus in neuroscience for measuring cognitive effort using EF tests (Kurzban et al. 2013).

#### Covariates

*Socioeconomic status (SES)*: Parents reported whether or not they had completed a tertiary educational degree, their perceived income adequacy, and their occupation, for which the corresponding value from the International Socio-Economic Index of Occupational Status (ISEI) was assigned.

*Further student characteristics: *Students reported their gender, age, and digital habits (mouse use and video gaming).^2^ Students also took a cognitive ability test that measures fluid intelligence using the standard version of the Raven’s Progressive Matrices Test (Raven and Court 1998).^3^

*Parenting styles and parental work hours: *Students reported how they perceived their parents’ levels of support or discipline. Parents also reported the number of hours they worked per week.

Table 3 in the appendix includes additional information on the definitions and measurement of the covariates.

### Analytical Plan

Some students had missing data due to incomplete or illegible surveys, particularly for parent-reported variables due to nonresponse on parent surveys. To avoid biased results from a complete-case analysis, we used multiple imputation with multilevel joint modeling to impute missing data. Using the *jomo.smc* function in the R package jomo (v.2.7.6; Quartagno and Carpenter 2023), we imputed missing data on all predictors (m = 10). After imputation, 34.4% of students were observed as living in a single-parent household (31.5% in Madrid, 38.5% in Berlin). Descriptive statistics of the covariates conditional on parental status are shown in Table 1.

**Table 1:** Descriptive statistics for dual- and single-parent households (*n* = 1359)*

	Percentage missing	Total	Dual-parent household	Single-parent household
*Gender*	3.7%	–	–	–
Girl	–	52.1%	52.6%	50.9%
Boy	–	47.9%	47.4%	49.1%
*City*	0.0%	–	–	–
Madrid	–	59.1%	61.7%	54.2%
Berlin	–	40.9%	38.3%	45.8%
*Parental tertiary education*	24.9%	–	–	–
No tertiary degree	–	49.0%	40.8%	64.6%
Tertiary degree	–	51.0%	59.2%	35.4%
*School type*	0.0%	–	–	–
Private	–	31.6%	36.0%	23.3%
Public	–	68.4%	64.0%	76.7%
*School neighborhood income quartile*	0.0%	–	–	–
1st	–	25.2%	22.2%	30.8%
2nd	–	26.4%	25.6%	28.0%
3rd	–	22.6%	22.2%	23.3%
4th	–	25.8%	30.0%	17.8%
*Age (months)*	2.6%	–	–	–
Mean	–	127.9	127.2	129.2
SD	–	7.0	6.6	7.5
*Raven’s Matrices score*	3.6%	–	–	–
Mean	–	24.2	24.7	23.4
SD	–	4.7	4.6	4.9
*Mouse use*	2.2%	–	–	–
Mean	–	1.2	1.2	1.2
SD	–	1.2	1.1	1.2
*Video gaming*	2.4%	–	–	–
Mean	–	2.1	2.0	2.2
SD	–	1.2	1.2	1.2
*Parental support*	19.5%	–	–	–
Mean	–	3.8	3.8	3.7
SD	–	0.5	0.5	0.6
*Parental discipline*	20.0%	–	–	–
Mean	–	2.1	2.3	1.9
SD	–	0.9	0.9	0.9
*Perceived income adequacy*	19.0%	–	–	–
Mean	–	3.4	3.5	3.2
SD	–	0.7	0.7	0.7
*ISEI*	20.9%	–	–	–
Mean	–	47.6	50.3	42.6
SD	–	17.6	16.9	17.9
*Mother or sole guardian work hours*	18.4%	–	–	–
Mean	–	1.1	1.1	1.1
SD	–	0.7	0.7	0.7
*Observations*	–	1359	892	467

* Counts, means, and standard deviations (SDs) are calculated as the mean values across the ten imputed datasets. *ISEI* International Socio-Economic Index of Occupational Status

Two-level random-intercept linear regression models were used to account for between-student variation in baseline real effort. The hierarchical models were run using the *lmer* function of the R package *lme4* (v.1.1.31) on each of the ten imputed datasets (Bates et al. 2023). The main regression models were also fit on the complete-case data for comparison (Table 4 in the appendix).^4^

## Results

**Fig. 1:**
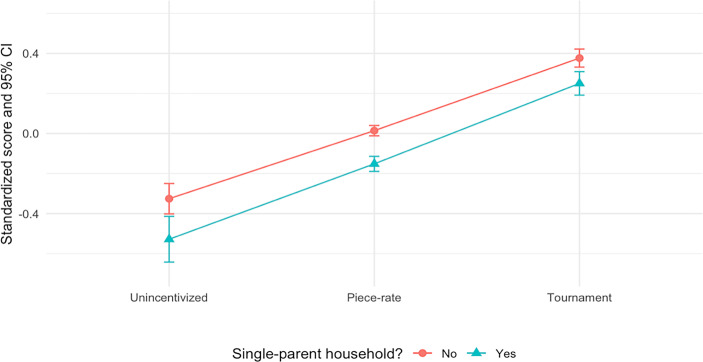
Average standardized score in real-effort task performance and 95% confidence intervals across incentive conditions, by single- and dual-parent household status

Figure 1 shows raw descriptive evidence. As can be seen, children who were living with both parents performed better on the real-effort tasks than children who were living with only one parent. The performance differential is about one-fifth of a standard deviation (SD) of the mean score. Moreover, the observed differences are significantly positive for children with two parents whatever the incentive condition (unincentivized, piece-rate, or tournament) under which they took the tests.

To further scrutinize the association between children’s cognitive effort and living in a single-parent household, we constructed five regression models that iteratively control for groups of variables. For simplicity, this analysis exclusively examines the piece-rate condition, given that (a) incentive conditions turned out not to moderate the absent-parent effect, and (b) all three real-effort tasks were performed for two rounds each in this condition. To isolate cognitive effort, model 1 controls for cognitive ability in the form of fluid intelligence, as well for technical proficiency as measured by frequency of mouse use and video gaming. The estimate shows a negative association between living in a single-parent household and cognitive effort (β = −0.116 SD, *p* = 0.001). Model 2 includes a set of variables aimed to comprehensively control for SES: parental education, occupational status, and perceived income adequacy. This is our preferred specification. As expected, accounting for SES reduces the estimated effect associated with single parenthood, though it remains significant and negative (β = −0.087 SD, *p* = 0.018). Model 3 includes environmental-level variables including school type and school–neighborhood income quartile. The single-parent effect remains unchanged (β = −0.086 SD, *p* = 0.019), indicating that school segregation plays little role in the single-parent effect on cognitive effort.

Addressing potential mediating variables, model 4 controls for the sole mother’s or sole father’s reported work hours to explore whether the effect of living in a single-parent household may be operating through mechanisms such as lack of parental engagement or parental stress. However, little to no movement is seen in the single-parent effect when parental working hours are included in the model (β = −0.087 SD, *p* = 0.017). Finally, model 5 controls for parenting styles and shows that heterogeneities in parental support or discipline may be contributing, if anything, only slightly to the single-parent effect on children’s effort (β = −0.076 SD, *p* = 0.042).

**Fig. 2:**
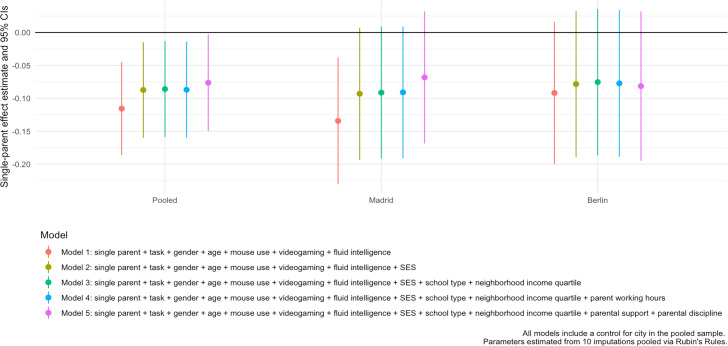
Single-parent household effect on child’s effort intensity, pooled and by city. *SES* socioeconomic status

In Fig. 2, we conduct the analysis separately for each city and find qualitatively similar results in both places. This indicates the robustness of our main findings in a macrosocial context. That being said, the estimated negative single-parent effect is slightly greater in magnitude in Madrid than in Berlin (estimates from our preferred model 2: β_Madrid_ = −0.093 SD, *p*_Madrid_ = 0.068; β_Berlin_ = −0.078 SD, *p*_Berlin_ = 0.166). Moreover, the uncertainty of the estimates is higher in Berlin, where the sample size is smaller, and we find even less evidence that the negative relationship between single-parent background and cognitive effort may be explained by parenting styles.

## Discussion

This brief report details the association between single-parent households and cognitive effort among fifth-graders in Berlin and Madrid, a crucial yet underresearched area in understanding educational disparities and other child outcomes. Our findings consistently demonstrate a negative association between living in a single-parent household and children’s cognitive effort, measured behaviorally through performance on real-effort tasks testing EF. Even though we caution against purely voluntaristic interpretations of effort (cf. Radl et al. 2026), this means that children living with only one parent tend to invest fewer mental resources to accomplish simple yet demanding tasks.

Specifically, we found that children from single-parent households performed significantly worse on cognitive effort tasks across all incentive conditions (unincentivized, piece-rate, and tournament). The negative association remained statistically significant even after parental education, occupational status, and perceived income adequacy were accounted for. The effect sizes—approximately one-fifth of a standard deviation of task performance scores unconditionally and one-tenth after conditioning on socioeconomic factors—are similar to previous estimates from family research examining related outcomes. Interestingly, school type and neighborhood income played a minimal role in explaining this effect, suggesting that the “single-parent effect” on cognitive effort is not substantially driven by school segregation.

Although there is a dearth of studies on the relationship between single parenthood and children’s cognitive effort, previous research has documented systematic associations between parenting behavior and children’s EF. Sosic-Vasic et al. (2017) found that for a European-based sample of school-aged children, high levels of parental involvement were associated with lower error rates on tasks measuring EF. Our analysis explored potential mediating mechanisms of the single parenthood–EF relationship, such as parental work hours and parenting styles. Although accounting for these factors slightly attenuated the observed effect, the negative relationship between single parenthood and cognitive effort largely remained significant. This suggests that while parenting behaviors may play some role, they do not explain the bulk of the observed differences in cognitive effort by family form.

The qualitative similarity of results in both Berlin and Madrid, despite minor differences in magnitude and statistical significance, signals a crucial robustness across distinct macrosocial contexts. This is particularly salient given that Germany and Spain possess different family and educational systems—for instance, Germany traditionally features earlier tracking in education and a stronger stratification, whereas Spain emphasizes a more comprehensive education until later ages. These differences may plausibly evoke distinct involvement patterns of parents when it comes to educational guidance and support of their children, but the cross-national consistency in our results indicates that the single-parent effect may be applicable to a broader population of fifth-graders beyond these two specific metropolitan centers with elevated shares of single parents.

Furthermore, the stability of our results under varying experimental incentive conditions, and after controlling for possible divergences in parental styles and levels of engagement, strengthens this generalizability. Substantively, these findings seem to indicate that the association between parental absence and poor cognitive effort depends more on the family structure itself than on other environmental factors. Because single parenthood per se tends to undermine children’s cognitive effort under varying environmental conditions, it appears as a relevant, independent source of variation for poor cognitive engagement among early adolescents, plausibly hindering their educational achievement.

A limitation of this study is the lack of information on the family dynamics that gave rise to single-parent households, including the duration of these situations. The implications for children’s cognitive effort may vary depending on whether parental separation, widowhood, or intentional single parenthood led to children growing up with only one parent. Because the timing of family events is unknown, it cannot be tackled in our empirical exercise either. Studies to come should address this potential source of heterogeneity.

This study contributes to the literature on adolescent development by providing empirical evidence, based on behavioral measures, that single parenthood is associated with diminished cognitive effort in children. Although the estimated single-parent effect on cognitive effort, at approximately −0.09 SD, is considered modest by conventional effect size guidelines, its consistency and statistical significance underscore its practical importance. Even a small deficit in cognitive effort at a crucial developmental stage, such as fifth grade, can have cumulative adverse effects on academic performance and future opportunities. Therefore, addressing this gap, even if seemingly small, remains a critical goal for educational and social policy interventions. Future research could delve deeper into the specific mechanisms that contribute to this reduced cognitive effort, such as chronic stress, loss of household resources, or the unique social–emotional experiences of children in single-parent families, to inform targeted interventions aimed at mitigating these disparities.

## Data Availability

Although data protection policies forbid sharing of the German part of the data, the Spanish part of the data will be publicly available (after expiration of the embargo period) on the data repository of the grant holder institution: 10.21950/DEDRIZ. Analysis code to replicate results for the Spanish subsample will also be available.

## Appendix

**Table 2:** Number and percentage of students whose schools belong to each sampling cluster, by city

City	Sampling cluster	Number	Percentage
*Madrid*	Private	27	3.36
	Public, 1st income quartile	110	13.68
	Public, 2nd income quartile	102	12.69
	Public, 3rd income quartile	129	16.04
	Public, 4th income quartile	67	8.33
	Semiprivate, 1st income quartile	93	11.57
	Semiprivate, 2nd income quartile	120	15.05
	Semiprivate, 3rd income quartile	50	6.22
	Semiprivate, 4th income quartile	105	13.06
*Berlin*	*Frühgymnasien*/early tracking	55	9.89
	Private	35	6.29
	Public, 1st income quartile	101	18.17
	Public, 2nd income quartile	137	24.64
	Public, 3rd income quartile	102	18.35
	Public, 4th income quartile	126	22.66

**Table 3:** Covariate definitions and measurement*

Variable group	Variable	Description/measurement	Scale
*Socioeconomic status*	Parent tertiary degree	Indicator for whether at least one parent completed a tertiary education	*0* *=* *No tertiary degree* *1* *=* *Tertiary degree*
	Income adequacy	Ordinal measure of parental self-assessment of how difficult it is to get by on current household income	*1* *=* *Very difficult* *2* *=* *Difficult* *3* *=* *Coping* *4* *=* *Living comfortably*
	Parental ISEI	ISEI based on reported occupation	Continuous
*Student background*	Gender	Indicator of student-reported gender	*0* *=* *Girl* *1* *=* *Boy*
	Age	Student age in months	Continuous
	Mouse use	Ordinal measure of the frequency of using a desktop computer with a mouse	*0* *=* *Never/almost never* *1* *=* *Once or twice a month* *2* *=* *Once or twice a week* *3* *=* *Every day/almost every day*
	Video gaming	Ordinal measure of the frequency of playing video games on any digital device	*0* *=* *Never* *1* *=* *<* *30* *min/day* *2* *=* *30* *min–1* *h/day* *3* *=* *1–2* *h/day* *4* *=* *2+ h/day*
–	Fluid intelligence	Number of correct responses on Raven’s Progressive Matrices (standard version, 5 min)	Continuous
*Parenting and parental labor supply*	Parental support	Ordinal measure of student-reported perceived parental support (*“Would you say your parent is very supportive or not that much?”)*	*1* *=* *Supports a little* *2* *=* *Supports sometimes* *3* *=* *Supports somewhat* *4* *=* *Supports a lot*
	Parental discipline	Ordinal measure of student-reported perceived parental discipline (*“Would you say your parent is strict regarding the things you need to do [for example, time for bed, time for homework, participation in extracurricular activities]?”*)	*1* *=* *A little strict* *2* *=* *Somewhat strict* *3* *=* *Quite strict* *4* *=* *Very strict*
	Parental work hours	Ordinal measure of parent-reported weekly hours worked	*0* *=* *Unemployed* *1* *=* *<* *20* *h/week* *2* *=* *20–34* *h/week* *3* *=* *35+ h/week*

***** For participants living in dual-parent households, the maximum level of both parents’ educational attainment, International Socio-Economic Index of Occupational Status (ISEI), and support/discipline was retained. Number of hours worked was indicated by the mother’s reported working hours

**Table 4:** Effect of single-parent household on effort intensity (piece-rate), Madrid and Berlin

	Imputed (All)	Complete (All)	Imputed (Madrid)	Complete (Madrid)	Imputed (Berlin)	Complete (Berlin)
*Single parent*	−0.09* (0.04)	−0.08 (0.05)	−0.09 (0.05)	−0.09 (0.06)	−0.08 (0.06)	−0.08 (0.08)
*Task: slider*	0.00 (0.02)	0.03 (0.03)	0.00 (0.03)	−0.01 (0.03)	0.00 (0.03)	−0.04 (0.05)
*Task: Simon*	−0.00 (0.02)	−0.05 (0.03)	0.00 (0.03)	−0.02 (0.03)	−0.00 (0.03)	−0.09 (0.05)
*City: Berlin*	−0.00 (0.04)	0.07 (0.05)	–	–	–	–
*Gender: Boy*	0.36*** (0.04)	0.36*** (0.04)	0.32*** (0.05)	0.35*** (0.05)	0.40*** (0.05)	0.39*** (0.08)
*Age (months)*	0.00 (0.00)	−0.00 (0.00)	0.00 (0.00)	0.00 (0.00)	0.00 (0.00)	−0.00 (0.01)
*Fluid intelligence (std.)*	0.23*** (0.02)	0.23*** (0.02)	0.20*** (0.02)	0.22*** (0.02)	0.27*** (0.03)	0.24*** (0.04)
*Mouse use*	0.06*** (0.01)	0.04* (0.02)	0.06** (0.02)	0.05* (0.02)	0.06** (0.02)	0.02 (0.03)
*Video gaming*	0.01 (0.01)	0.03 (0.02)	0.06** (0.02)	0.05* (0.02)	−0.07** (0.02)	−0.04 (0.03)
*Parent tertiary degree*	0.10* (0.04)	0.14** (0.05)	0.13* (0.06)	0.16* (0.06)	0.07 (0.07)	0.13 (0.10)
*Parental ISEI (std.)*	0.04 (0.02)	0.05 (0.03)	0.05 (0.03)	0.03 (0.03)	0.03 (0.04)	0.06 (0.05)
*Income adequacy (std.)*	−0.02 (0.02)	−0.04 (0.02)	−0.02 (0.03)	−0.02 (0.03)	−0.02 (0.03)	−0.09* (0.04)
*SD—subject*	0.51	0.51	0.51	0.52	0.50	0.50
*SD—observation*	0.80	0.80	0.80	0.78	0.80	0.82
*Subjects*	1359	921	803	655	556	266
*Observations*	7966	5404	4714	3843	3252	1561

****p* ≤ 0.001, ***p* ≤ 0.01, **p* ≤ 0.05

All models are two-level hierarchical models grouped at the student level. *std.* standardized, *ISEI* International Socio-Economic Index of Occupational Status, *SD* standard deviation

## Footnotes

1 Except for one early-tracking secondary school in Berlin, all fifth-graders in both cities attended primary school.

2 These controls are included because children’s familiarity with computers and video games would influence their performance on the computerized behavioral tasks independently of their propensity to exert effort. The frequency with which they use a computer mouse is particularly important when it comes to the mouse-based Slider task.

3 Although tasks were designed to minimize the impact of cognitive ability, we controlled for IQ to minimize any residual influence that intelligence can still exert on task performance.

4 The coefficient for living in a single-parent household is remarkably stable independent of the treatment of missing data, although the loss of statistical power results in lower precision of the estimate in smaller samples.

## References

[CR1] Ahrons, Constance R. 2007. Family ties after divorce: Long-term implications for children. *Family Process* 46(1): 53–65. 17375728 10.1111/j.1545-5300.2006.00191.x

[CR2] Amato, Paul R. 2005. The impact of family formation change on the cognitive, social, and emotional well-being of the next generation. *Future of Children* 15(2): 75–96. 16158731 10.1353/foc.2005.0012

[CR3] Amato, Paul R. 2010. Research on divorce: continuing trends and new developments. *Journal of Marriage and Family* 72: 650–666.

[CR4] Andrews, Kirsten, Judith R. Dunn, Heather Prime, Marc Jambon, Dillon Browne and Jennifer M. Jenkins. 2021. Effects of household chaos and parental responsiveness on child executive functions: a novel, multi-method approach. *BMC Psychology* 9: 1–14. 34548106 10.1186/s40359-021-00651-1 PMC8456676

[CR5] Anthony, Christopher J., James C. DiPerna and Paul R. Amato. 2014. Divorce, approaches to learning, and children’s academic achievement: A longitudinal analysis of mediated and moderated effects. *Journal of School Psychology* 52(3): 249–261. 24930818 10.1016/j.jsp.2014.03.003

[CR6] Arnsten, Amy F. T. 2009. Stress signalling pathways that impair prefrontal cortex structure and function. *Nature Reviews Neuroscience* 10: 410–422. 19455173 10.1038/nrn2648 PMC2907136

[CR7] Bates, Douglas, Martin Mächler, Benjamin Bolker and Steve Walker. 2023. Lme4: Linear Mixed-Effects Models Using Eigen and S4. https://github.com/lme4/lme4/.

[CR8] Bloome, Deirdre. 2017. Childhood family structure and intergenerational income mobility in the United States. *Demography* 54: 541–569. 28315158 10.1007/s13524-017-0564-4 PMC5858195

[CR9] Bzostek, Sharon H. and Lawrence M. Berger. 2017. Family structure experiences and child socioemotional development during the first nine years of life: Examining heterogeneity by family structure at birth. *Demography* 54(2): 513–540. 28299560 10.1007/s13524-017-0563-5 PMC5948107

[CR10] Carlson, Marcia J. and Mary E. Corcoran. 2001. Family Structure and Children’s Behavioral and Cognitive Outcomes. *Journal of Marriage and Family* 63: 779–792.

[CR11] Cavanagh, Shannon E. and Paula Fomby. 2019. Family Instability in the Lives of American Children. *Annual Review of Sociology* 45: 493–513. 10.1146/annurev-soc-073018-022633 PMC7388657 32728311

[CR12] Cespón, Jesús, Santiago Galdo-Álvarez and Fernando Díaz. 2016. Cognitive Control Activity Is Modulated by the Magnitude of Interference and Pre-Activation of Monitoring Mechanisms. *Scientific Reports* 6(1): 39595. 27995983 10.1038/srep39595 PMC5171494

[CR13] Cherlin, Andrew J., Frank F. Furstenberg Jr., P. Lindsay Chase-Lansdale, Kathleen E. Kiernan, Philip K. Robins, Donna Ruane Morrison and Julien O. Teitler. 1991. Longitudinal studies of effects of divorce on children in Great Britain and the United States. *Science* 252(5011): 1386–1389. 2047851 10.1126/science.2047851

[CR14] Coe, Jillian L., Patrick T. Davies and Melissa L. Sturge-Apple. 2017. Family instability and young children’s school adjustment: callousness and negative internal representations as mediators. *Child Development* 89: 1193–1208. 28369999 10.1111/cdev.12793 PMC5617759

[CR15] Dang, Jianping, Kevin M. King and Michael Inzlicht. 2020. Why are self-report and behavioral measures weakly correlated? *Trends in Cognitive Sciences* 24(4): 267–269. 32160564 10.1016/j.tics.2020.01.007 PMC7977810

[CR46] de Lange, Marloes, Jaap Dronkers and Maarten H. J. Wolbers. 2014. Single-parent family forms and children’s educational performance in a comparative perspective: effects of school’s share of single-parent families. *School Effectiveness and School Improvement* 25(3):329-350. 10.1080/09243453.2013.809773

[CR16] Demo, David H. and Alan C. Acock. 1996. Family structure, family process, and adolescent well-being. *Journal of Research on Adolescence* 6(4): 457–488.

[CR17] Finegood, Eric D. and Clancy Blair. 2017. Poverty, Parent Stress, and Emerging Executive Functions in Young Children. In *Parental Stress and Early Child Development*, eds. Kirby Deater-Deckard and Robin Panneton. Cham: Springer.

[CR18] Foley, William and Jonas Radl. 2024. Parenting Practices and Children’s Cognitive Effort: A Laboratory Study. *The Journal of Early Adolescence* 45(4): 481–507.

[CR19] Ganong, Lawrence, Marilyn Coleman and Luke T. Russell. 2015. Children in Diverse Families. In *Handbook of Child Psychology and Developmental Science*, 7th edition, ed. Richard M. Lerner, 133–174. Hoboken: John Wiley & Sons.

[CR20] Garriga, Anna and Fulvia Pennoni. 2022. The causal effects of parental divorce and parental temporary separation on children’s cognitive abilities and psychological well-being according to parental relationship quality. *Social Indicators Research* 161(2): 963–987.

[CR21] Gill, David and Victoria Prowse. 2019. Measuring Costly Effort Using the Slider Task. *Journal of Behavioral and Experimental Finance *21: 1–9.

[CR22] Hadfield, Kristin, Mary Amos, Michael Ungar, Josée Gosselin and Lawrence Ganong. 2018. Do changes to family structure affect child and family outcomes? A systematic review of the instability hypothesis. *Journal of Family Theory & Review* 10(1): 87–110.

[CR23] Härkönen, Juho, Fabrizio Bernardi and Diederik Boertien. 2017. Family Dynamics and Child Outcomes: An Overview of Research and Open Questions. *European Journal of Population* 33: 163–184. 30976231 10.1007/s10680-017-9424-6 PMC6240988

[CR24] Hefer, Christian and Gesine Dreisbach. 2016. The Motivational Modulation of Proactive Control in a Modified Version of the AX-Continuous Performance Task: Evidence from Cue-Based and Prime-Based Preparation. *Motivation Science* 2: 116–134.

[CR25] Kalmijn, Matthijs, Anne Loeve and Dorien Manting. 2007. Income dynamics in couples and the dissolution of marriage and cohabitation. *Demography* 44(1): 159–179. 17461341 10.1353/dem.2007.0005

[CR26] Kim, Hyunjoon S. 2011. Consequences of Parental Divorce for Child Development. *American Sociological Review* 76(3): 487–511.

[CR27] Kurzban, Robert, Angela L. Duckworth, Joseph W. Kable and Justus Myers. 2013. An opportunity cost model of subjective effort and task performance. *Behavioral and Brain Sciences* 36(6): 661–679. 24304775 10.1017/S0140525X12003196 PMC3856320

[CR28] Mathôt, Sebastiaan, Daniel Schreij and Jan Theeuwes. 2012. OpenSesame: An Open-Source, Graphical Experiment Builder for the Social Sciences. *Behavior Research Methods* 44: 314–324. 22083660 10.3758/s13428-011-0168-7 PMC3356517

[CR32] McBride Murry, Velma and Melissa A. Lippold. 2018. Parenting practices in diverse family structures: Examination of adolescents’ development and adjustment. *Journal of Research on Adolescence* 28(3): 650–664. 30515943 10.1111/jora.12390

[CR29] McLanahan, Sara and Gary D. Sandefur. 1994. *Growing Up with A Single Parent. What Hurts, What Helps*. Cambridge: Harvard University Press.

[CR30] McLanahan, Sara, Laura Tach and Daniel Schneider. 2013. The causal effects of father absence. *Annual Review of Sociology *39: 399–427. 10.1146/annurev-soc-071312-145704 PMC3904543 24489431

[CR31] Miyake, Akira and Naomi P. Friedman. 2012. The nature and organization of individual differences in executive functions: Four general conclusions. *Current Directions in Psychological Science *21(1): 8–14. 22773897 10.1177/0963721411429458 PMC3388901

[CR33] Neuenschwander, Regula, Patrizia Cimeli, Marianne Röthlisberger and Claudia M. Roebers. 2013. Personality Factors in Elementary School Children: Contributions to Academic Performance over and above Executive Functions? *Learning and Individual Differences* 25: 118–125.

[CR34] Quartagno, Matteo and James R. Carpenter. 2023. Jomo: A Package for Multilevel Joint Modelling Multiple Imputation. https://CRAN.R-project.org/package=jomo.

[CR35] Radl, Jonas, Leire Salazar and Héctor Cebolla Boado. 2017. Does Living in a Fatherless Household Compromise Educational Success? A Comparative Study of Cognitive and Non-cognitive Skills. *European Journal of Population* 33: 217–242. 28490829 10.1007/s10680-017-9414-8 PMC5400797

[CR36] Radl, Jonas, William Foley, Lukasz K. Kröger, Paula Lorente, Alicia Palacios-Abad, Heike Solga, Jan Stuhler and Michael Swarr. 2026. The Social Origins of Effort: How Incentives Reduce Socioeconomic Disparities among Children. *American Sociological Review* 91: 89–122.

[CR37] Raley, Ruth Kelly and Megan M. Sweeney. 2020. Divorce, Repartnering, and Stepfamilies: A Decade in Review. *Journal of Marriage and Family* 82: 81–99. 38283127 10.1111/jomf.12651 PMC10817771

[CR38] Raven, John Carlyle and John H. Court. 1998. *Raven’s Progressive Matrices and Vocabulary Scales*. Oxford: Oxford Psychologists Press.

[CR39] Ribar, David C. 2015. Why marriage matters for child wellbeing. *Future of Children* 25(2): 11–27.

[CR40] Roskam, Isabelle, Marie Stievenart, Jean-Christophe Meunier and Marie-Pascale Noël. 2014. The development of children’s inhibition: Does parenting matter? *Journal of Experimental Child Psychology* 122: 166–182. 24607865 10.1016/j.jecp.2014.01.003

[CR41] Smith, Thomas E. 1990. Parental Separation and the Academic Self-Concepts of Adolescents: An Effort to Solve the Puzzle of Separation Effects. *Journal of Marriage and Family* 52(1): 107–118.

[CR42] Sosic-Vasic, Zrinka, Julia Kröner, Stefan Schneider, Nebojša Vasic, Manfred Spitzer and Judith Streb. 2017. The Association between Parenting Behavior and Executive Functioning in Children and Young Adolescents. *Frontiers in Psychology* 8: 472. 28424644 10.3389/fpsyg.2017.00472 PMC5371664

[CR43] Sun, Yongmin and Yuanzhang Li. 2002. Children’s well-being during parents’ marital disruption process: A pooled time-series analysis. *Journal of Marriage and Family* 64(2): 472–488.

[CR44] Suor, Jennifer H., Melissa L. Sturge-Apple, Patrick T. Davies, Dante Cicchetti and Leah G. Manning. 2015. Tracing differential pathways of risk: associations among family adversity, cortisol, and cognitive functioning in childhood. *Child Development* 86: 1142–1158. 26081792 10.1111/cdev.12376 PMC4683120

[CR45] Tartari, Melissa. 2015. Divorce And the Cognitive Achievement of Children. *International Economic Review *56(2): 597–645*.*

